# Evaluation of cardiac parameters and other safety outcomes of brolucizumab treatment in patients with neovascular age‐related macular degeneration

**DOI:** 10.1002/prp2.897

**Published:** 2022-03-18

**Authors:** Nadia Zakaria, Nicolas Guerard, Andres Emanuelli, Pravin Dugel, Jen Watts, Melissa Liew, Margarita Gekkieva, Markus Hinder

**Affiliations:** ^1^ Novartis Institutes for BioMedical Research Translational Medicine Cambridge Massachusetts USA; ^2^ Global Drug Development Novartis Pharma AG Basel Switzerland; ^3^ Emanuelli Research and Development Center Arecibo Puerto Rico USA; ^4^ IVERIC bio New York New York USA; ^5^ Novartis Pharmaceuticals Corporation East Hannover New Jersey USA; ^6^ Novartis Institutes for BioMedical Research Translational Medicine Basel Switzerland

**Keywords:** anti‐VEGF, brolucizumab, cardiovascular, intravitreal, nAMD

## Abstract

This was a prospective, single‐dose, single‐arm, open‐label, non‐randomized, multicenter clinical study to determine cardiovascular safety after a single brolucizumab 6 mg intravitreal injection in neovascular age‐related macular degeneration patients (*N* = 14). Electrocardiogram (ECG) data were collected at different time points using 12‐lead Holter and standard ECG, and patients were followed up to 8 days (end of study) for any signs of ocular and non‐ocular adverse events (AEs). No clinically meaningful changes were observed in cardiac parameters. No patient had a ≥30 msec change from baseline in heart rate–corrected QT using Fridericia's formula (QTcF), and no patient had a new QTcF value of ≥450 msec between 20 and 24 h after treatment. No deaths or serious AEs were reported during the study period. These results are in line with the absence of new cardiovascular safety signal based on the ECG recordings collected over the first year of the pivotal studies performed with brolucizumab in DME.

**Trial Registration:** ClinicalTrials.gov identifier: NCT03954626.

## Graphical abstract

No clinically significant changes were observed in cardiac parameters after administration of a single 6mg dose of brolucizumab intravitreal injection. The findings are in line with expected outcomes for brolucizumab due to its small molecular size which leads to a faster systemic clearance and a 4‐fold lower systemic exposure compared with other anti‐VEGFs.

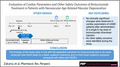

**Abbreviations**
AEs: adverse events
CV: cardiovascular
DME: diabetic macular edema
ECG: Electrocardiogram
EOS: end of study
GPP3: Good Publication Practice
HR: heart rate
IVT: intravitreal
nAMD: neovascular age‐related macular degeneration
QTcF: QT using Fridericia's formula
RVO: retinal vein occlusion
VEGF: vascular endothelial growth factor

**What is already known about this subject**

- Systemic adverse events have periodically been raised as a concern for anti–vascular endothelial growth factor (VEGF) therapy in neovascular age‐related macular degeneration (nAMD) patients, especially because they present with comorbidities, such as diabetes and hypertension, often associated with advanced age.
- Pivotal clinical studies have confirmed the positive benefit risk profile of ocular anti‐VEGF therapy and the number of reported systemic/cardiovascular incidences with these anti‐VEGFs are low overall.
- No previous studies have specifically described the effect of ocular anti‐VEGFs on electrocardiogram (ECG)‐assessed cardiac parameters.

**What this study adds**

- This is to our knowledge the first clinical study to describe the effect of intravitreal anti‐VEGF treatment (brolucizumab 6 mg) on multiple 12‐lead ECG‐derived parameters in nAMD patients. The results from this study further corroborate previously published safety results with brolucizumab in nAMD.
- No clinically significant changes were observed in cardiac parameters of nAMD patients after administration of a single 6 mg dose of brolucizumab intravitreal injection.

## 1 INTRODUCTION

Neovascular age‐related macular degeneration (nAMD) is the leading cause of vision loss among elderly people in developed countries^1^, ^2^ accounting for ~90% of AMD‐associated vision loss.^2^ For over a decade anti‐vascular endothelial growth factor (VEGF) therapy has been the clinical standard‐of‐care for nAMD.^3^, ^4^, ^5^

A wealth of evidence supports the efficacy and safety of anti‐VEGFs in the treatment of nAMD.^3^, ^4^, ^5^ However, it is important to note that VEGF inhibition may potentially induce systemic adverse effects that could be serious, especially for patients with diabetes or the elderly (including patients with nAMD) who are at an increased risk for cardiovascular (CV) adverse events (AEs).^6^, ^7^ Pivotal clinical studies have confirmed the positive benefit risk profile of ocular anti‐VEGF therapy and the number of reported systemic/cardiovascular incidences with these anti‐VEGFs are low overall.^6^, ^7^ Furthermore, no significant association between ocular intravitreal (IVT) anti‐VEGF administration and CV concerns has been established to date.^7^, ^8^, ^9^

Brolucizumab, a single‐chain antibody fragment, has recently been approved for the treatment of nAMD in the USA, Canada, Australia, Japan, and the EU, based on the confirmatory results from the Phase III HAWK and HARRIER trials.^10^ The unique molecular design of brolucizumab allows for a concentrated molar dosing in one IVT injection, effective tissue penetration, and increased duration of action.^10^, ^11^, ^12^, ^13^ Based on currently available pre‐clinical and clinical data, brolucizumab shows no potential to cause cardiac liabilities, especially due to its low molecular weight and high affinity for the VEGF‐A receptor, which enables a rapid and more effective target tissue penetration, resulting in very low systemic exposure and faster systemic clearance.^12^, ^13^

Patients with nAMD on average are elderly and often have accompanying concomitant diseases, including CV comorbidities.^6^, ^7^ These comorbidities are also noticeably higher in other indications, e.g., diabetic macular edema (DME)^7^ and retinal vein occlusion (RVO),^7^ for which brolucizumab is currently under development. Here, we aimed to investigate the CV safety and effects on ECG‐derived parameters after a single IVT injection of brolucizumab 6 mg in patients with nAMD.

## 2 MATERIALS AND METHODS

This was a prospective, single dose, single‐arm, open‐label, non‐randomized multicenter clinical study to collect 12‐lead ECG data after a single IVT injection of brolucizumab 6 mg in patients with nAMD. The study was conducted at four sites in US (1001_Emanuelli Research and Development Center, LLC, Arecibo; 1002_Retinal Research Institute, LLC, Phoenix; 1003_Intergrated Clinical Research, Abilene; 1004_MedEye Associates). The study protocol, the patient's information, and consent form were approved by the Advarra (an Independent Ethics Committee). The study number at Advarra is Pro00033738; study was approved to the protocol allowed limit of 10–15 patients. The study adhered to the Declaration of Helsinki and was reviewed by the Independent Ethics Committees or Institutional Review Board for each center. Written informed consent was obtained from each patient before screening. The detailed study protocol is available in the File S1.

Fourteen patients with nAMD (aged ≥50 years) were enrolled and received a single IVT injection of brolucizumab 6 mg during the treatment phase. Triplicate 12‐lead ECG recordings were performed at screening ~2 h prior to the brolucizumab IVT injection on Day 1 after the conclusion of Holter monitoring on Day 3. Twelve‐lead Holter ECG recording was initiated ~1 h prior to the brolucizumab IVT injection and ended at 48 h post‐IVT. At the end of study (EOS) visit on Day 8, patients were contacted via telephone and information about AEs and concomitant medications collected. Additionally, a safety follow‐up call was performed on Day 31. The other safety assessments were standard for this indication/patient population.

The primary objective of the study was to evaluate 12‐lead ECG‐derived parameters after a single IVT injection of brolucizumab 6 mg in patients with nAMD by measuring the incidence between 20‐ and 24 h post‐injection of clinically relevant treatment‐emergent changes in heart rate (HR), PR, QRS, and HR‐corrected QT using Fridericia's formula (QTcF) interval (msec). The secondary objective was to evaluate the safety outcomes which included analysis of vital signs (at baseline, Day 1, and Day 3) and any ocular and non‐ocular AEs (including clinically relevant ECG abnormalities) until the EOS. There were no formal hypotheses for this study and all statistical analyses were descriptive in nature.

## 3 RESULTS

### 3.1 Baseline and demographic characteristics

All 14 patients with nAMD who enrolled across 3 sites in the US completed the study. At baseline, their median age was 77.0 years; all enrolled patients were Caucasian with 12 (86%) of Hispanic or Latino ethnicity; 8 (57%) were female. Mean systolic blood pressure, diastolic blood pressure, and HR of all patients were within normal range at baseline as per the inclusion criteria (Table 1). The enrolled patients presented with a medical history of hypertension (*n* = 5), hypercholesterolemia (*n* = 6), diabetes (*n* = 2), and hyperlipidemia (*n* = 1); however, no patient received medication that could have an effect on their QT interval at study start or during the study.

**TABLE 1:** Demographics and baseline characteristics

Characteristics	Brolucizumab 6 mg *N* = 14
Age (years)	
Mean (SD)	75.7 (6.57)
Gender, *n* (%)	
Female	8 (57.1)
Predominant race, *n* (%)	
Caucasian	14 (100.0)
Ethnicity, *n* (%)	
Hispanic or Latino	12 (85.7)
Systolic blood pressure (mmHg), mean (SD)	132.9 (5.27)
Diastolic blood pressure (mmHg), mean (SD)	76.3 (9.15)
Pulse rate (beats/min), mean (SD)	72.0 (9.55)

Note

Safety analysis set.

Abbreviations: *n*, number of patients in the treatment group; *N*, total number of patients tested; SD, standard deviation.

### 3.2 Primary outcomes

The 12‐lead Holter ECG provided comprehensive ECG data starting ~1 h before IVT injection to 48 h after, capturing the approximate time at which maximum systemic concentration was expected (median *T*_max_ = ~24 h post‐dose).^14^ The mean values for the Holter ECG‐assessed cardiac parameters, including HR, PR, QRS, and QTcF intervals, remained within normal ranges at all time‐points (Table 2) and no notable changes from baseline were observed for these parameters 20–24 h post‐treatment with 6 mg brolucizumab. Furthermore, no patient had a ≥30 or ≥60 msec change from baseline in QTcF and no patient had a new QTcF value of ≥450 msec 20–24 h post‐treatment (Table 3).

**TABLE 2:** Descriptive statistics of 12‐lead ECG parameters by visit/time

Assessment type	Visit/time	Statistics	Brolucizumab 6 mg (*N* = 14)			
			PR interval (msec)	QRS duration (msec)	QTcF interval (msec)	HR (beats/minute)
Holter ECG^a^	Baseline	Mean (SD)	175.4 (22.58)	89.0 (11.96)	419.0 (16.38)	72.2 (11.85)
		Median	169.0	88.0	418.0	71.2
		Range	129–223	75–125	388–444	55–96
	20 h Post‐injection	Mean (SD)	165.7 (25.36)	88.4 (11.43)	412.2 (14.28)	82.8 (15.24)
		Median	167.8	86.5	411.0	80.3
		Range	126–204	76–123	393–441	51–104
	22 h Post‐injection	Mean (SD)	170.2 (23.01)	88.7 (11.60)	417.4 (20.56)	78.9 (10.53)
		Median	167.5	87.0	420.2	78.3
		Range	132–208	73–123	371–444	62–94
	24 h Post‐injection	Mean (SD)	175.3 (28.26)	89.5 (10.88)	419.0 (17.71)	75.9 (10.25)
		Median	175.0	88.8	422.2	78.5
		Range	134–232	76–123	383–442	61–93
Standard ECG^b^	Baseline	Mean (SD)	182.5 (18.92)	94.0 (10.60)	419.2 (14.61)	68.8 (12.51)
		Median	175.5	90.8	417.3	70.0
		Range	160–217	80–125	397–448	49–96
	48 h Post‐injection	Mean (SD)	181.2 (23.93)	92.5 (12.03)	419.6 (18.97)	69.6 (11.83)
		Median	179.3	87.8	415.8	69.5
		Range	143–223	82–130	397–450	52–99

Note

Safety analysis set.

Abbreviations: ECG, electrocardiogram; HR, heart rate; IVT, intravitreal; *N*, total number of patients; R, replicates; SD, standard deviation.

^a^, ^b^The mean of the triplicate ECG values (*R* = 3) was calculated for each patient at each timepoint.

^a^ Triplicate 12‐lead Holter ECGs were collected at baseline, 20, 22, 24 h post‐injection. Baseline for 12‐lead Holter ECG parameters was defined at Day 1, 1 h prior to brolucizumab IVT injection.

^b^ Triplicate 12‐lead ECGs were collected at screening, baseline, and 48 h after injection. Baseline for 12‐lead ECG parameters was defined at Day 1, 2 h prior to the brolucizumab IVT injection.

**TABLE 3:** Descriptive statistics for 12‐lead Holter ECG QTcF parameter thresholds by visit/time

Visit/time	Brolucizumab 6 mg (*N* = 14)				
	Mean QTcF interval (msec) % (n/m)			Mean change in QTcF interval from baseline (msec) % (n/m)	
	>450	>480	>500	>=30	>=60
Baseline	0.0 (0/14)	0.0 (0/14)	0.0 (0/14)	—	—
20 h post‐injection	0.0 (0/14)	0.0 (0/14)	0.0 (0/14)	0.0 (0/14)	0.0 (0/14)
22 h post‐injection	0.0 (0/14)	0.0 (0/14)	0.0 (0/14)	0.0 (0/14)	0.0 (0/14)
24 h post‐injection	0.0 (0/14)	0.0 (0/14)	0.0 (0/14)	0.0 (0/14)	0.0 (0/14)

Note

Safety analysis set.

The mean of the triplicate Holter ECG values (*R* = 3) was calculated for each patient at each timepoint. Baseline for 12‐lead Holter ECG parameters was defined at Day 1, 1 h prior to the brolucizumab IVT injection.

Abbreviations: ECG, electrocardiogram; IVT, intravitreal; m, total number of patients with a value for a specific categorical variable; *n*, Number of patients who are at the corresponding category; *N*, total number of patients in the treatment group; *R*, replicates.

No clinically significant changes from baseline were noted in PR, QRS, QTcF, and HR when assessed using a standard 12‐lead ECG, which was consistent with the Holter ECG findings (Table 2).

### 3.3 Secondary outcomes

There were no deaths or non‐ocular AEs reported during the study or the follow‐up period (Day 31). No clinically relevant changes in vital signs or ECG abnormalities were observed during the study. Of the 14 enrolled patients, 1 patient experienced an ocular AE (increased intraocular pressure) of moderate severity, possibly related to study treatment. This event was transient (lasting for 1 min) and resolved without sequelae after a paracentesis was performed.

## 4 DISCUSSION

The findings of this study showed that no clinically relevant changes on 12‐lead Holter ECG or standard 12‐lead ECG derived parameters were observed within 48 h following a single IVT injection of brolucizumab 6 mg in patients with nAMD. Furthermore, no significant safety concerns were identified during the study.

Systemic AEs have periodically been raised as a concern for anti‐VEGF therapy in nAMD patients, especially since they present with comorbidities, such as diabetes and hypertension, often associated with advanced age. Biotherapeutics, such as anti‐VEGF monoclonal antibodies or antibody fragments, are not expected to have any cardiac liabilities; therefore, a thorough cardiac safety assessments are generally not required for these drugs.^15^

While a large body of evidence supports the safety of ocular anti‐VEGFs, such as ranibizumab and aflibercept, in nAMD with no increased risk of SAEs particularly, CV related AEs.^3^, ^4^, ^5^, ^7^, ^8^, ^9^ Georgakopoulos et al. confirmed that a single dose of aflibercept did not affect biomarkers associated with CV risks (homocysteine, total cholesterol, triglycerides, HDL‐c, LDL‐c and CRP) in AMD patients.^16^ Furthermore, the safety data from real world population‐based studies^17^, ^18^ on intravitreal anti‐VEGFs agents (ranibizumab, aflibercept and bevacizumab) in patients with AMD, found no consistent evidence that intravitreal anti‐VEGF therapy was associated with increased risk of stroke, MI, or death. Drugs in general, may increase the risk for cardiovascular events via effects on the vascular tone (e.g., hypertension), rheological effects (e.g., prothrombotic effects) or by affecting cardiac electrophysiology.^19^ Our study investigated over a 48‐h time period the potential electrophysiological effects (depolarization, conduction and repolarization) of intravitreal brolucizumab by Holter monitoring. No previous studies have specifically described the effect of these ocular anti‐VEGFs on the ECG–assessed cardiac parameters.

The new age brolucizumab, a recently approved anti‐VEGF drug for nAMD indication, by virtue of its unique engineered design and smallest molecular weight (26 kDa) compared with other anti–VEGF molecules, allows for more effective retinal penetration and thereby minimal subsequent systemic exposure.^12^, ^13^ Furthermore, owing to its shorter half‐life and rapid systemic clearance, brolucizumab does not accumulate in the systemic circulation after repeated monthly injections.^10^, ^11^, ^12^, ^13^ Preclinical and clinical studies have also confirmed no known systemic safety issues with brolucizumab.^10^, ^11^, ^12^, ^13^ Furthermore, recent real‐world evidences of brolucizumab treatment in nAMD patients did not report any new safety signals specific to CV events; however, none of them specifically investigated any ECG‐related cardiac parameters.^20^, ^21^

This present study is the first to describe the effect of intravitreal anti‐VEGF treatment (brolucizumab 6 mg) on multiple 12‐lead ECG‐derived parameters in nAMD patients. The results confirmed that no clinically relevant cardiac abnormalities were observed with brolucizumab. ECG‐derived parameters including HR, PR, QRS, and QTcF intervals, which represent conduction and repolarization stages of cardiac cycle, showed no clinically relevant changes on ECG recordings within 48 h, following brolucizumab treatment. No patient had reported an increase in QTcF interval >450 msec or increase of 30 msec from baseline. No clinically significant changes in vital signs were observed, except for high systolic blood pressure in 1 patient (140 mmHg) on Day 3. These safety findings based on cardiac parameters, will be further corroborated in the large pivotal studies on brolucizumab safety and efficacy in DME population (publication is currently underway; 2021).

### 4.1 Limitations

This study was non‐randomized, limited by a small sample size, and lack of control group. Furthermore, the time to maximum concentration (*T*_max_) of brolucizumab was estimated based on the previous pharmacokinetic assessments, nevertheless, the continuous ECG monitoring over 48 h ensured a comprehensive assessment of *T*_max_ in all patients.

## 5 CONCLUSIONS

To conclude, it was a small descriptive study to prospectively evaluate any possible short‐term effect of a single intravitreal injection of brolucizumab in cardiovascular risk, specifically in terms of ECG‐derived cardiac parameters. The findings of the study were in line with the expected outcomes for brolucizumab due to its small molecular size which leads to a faster systemic clearance and a 4‐fold lower systemic exposure compared with other anti‐VEGFs.^12^, ^13^ No other safety concerns were identified during the study. With a better durability of effect and lower systemic exposure brolucizumab can reduce the burden of frequent injections (as compared with other anti‐VEGF treatments) and help physicians to optimize treatment regimens, especially for nAMD patients with CV comorbidities. Furthermore, these results are in line with the absence of new cardiovascular safety signal based on the ECG recordings collected over the first year of the pivotal studies performed with brolucizumab in DME (publication is currently underway; 2021).

## DISCLOSURE

NZ, NG, JW, ML, MG, and MH are employees of Novartis; PD is an employee and shareholder of IVERIC bio, NY, USA; AE is the principal investigator of Novartis, Novartis Institute for Biomedical Research, Regeneron, Genentech/Roche, Apellis Pharmaceuticals, and Adverum Biotechnologies.

## AUTHOR CONTRIBUTIONS

NG, MH, NZ, MG, ML, JW were involved the conception and design of the studies included in this report; JW, NG, NZ, PD and AE were responsible for the acquisition of the data; NZ, NG, MH, ML, MG, JW were all involved in the analysis and interpretation of the results of the studies. All authors collaborated on the writing of the manuscript and made the decision to submit the manuscript for publication.

## ETHICS STATEMENT

The study adhered to the Declaration of Helsinki and was reviewed by the Independent Ethics Committees or Institutional Review Board for each center. Written informed consent was obtained from each patient before screening. The detailed study protocol is available in the File S1.

## Supporting information

File S1

## Data Availability

The data that support the findings of this study are available from the corresponding author upon reasonable request.
