# Supplementary material for: Evaluation of cardiac parameters and other safety outcomes of brolucizumab treatment in patients with neovascular age‐related macular degeneration
Source: Pharmacol Res Perspect. 2022 Mar 18;10(2):e00897. doi: 10.1002/prp2.897 (PMC8931501; doi:10.1002/prp2.897)
Supplement: Supplementary file 1 — File S1 [file PRP2-10-e00897-s001.docx]

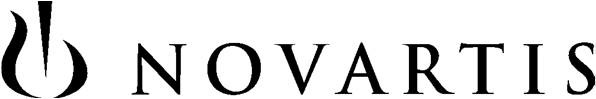


Novartis Institutes for BioMedical Research

RTH258/brolucizumab

Clinical Trial Protocol CRTH258A2309

**A Single-Arm, Open-Label, Multicenter, Phase IIIb Study to Collect Safety and Electrocardiogram Data on Brolucizumab 6 mg Intravitreal Treatment in Patients with Neovascular Age-Related Macular Degeneration**

Document type: Clinical Trial Protocol EUDRACT number: N/A

Version number: 00 (Original Protocol) Clinical trial phase: IIIb

Release date: 13-Mar-2019

Property of Novartis Confidential

May not be used, divulged, published, or otherwise disclosed without the consent of Novartis

**Clinical Trial Protocol Template Version 2.0 (01-Aug-2018)**

## Table of contents

Table of contents [2](#_bookmark0)

List of tables [4](#_bookmark1)

List of figures [4](#_bookmark1)

List of abbreviations [5](#_bookmark2)

Glossary of terms [8](#_bookmark3)

Protocol summary [10](#_bookmark4)

1. Introduction [12](#_bookmark5)
   1. Background [12](#_bookmark5)
   2. Purpose [13](#_bookmark6)
2. Objectives and endpoints [14](#_bookmark7)
3. Study design [14](#_bookmark7)
4. Rationale [15](#_bookmark8)
   1. Rationale for study design [15](#_bookmark8)
   2. Rationale for dose/regimen and duration of treatment [16](#_bookmark9)
   3. Rationale for choice of control drugs [16](#_bookmark9)
   4. Purpose and timing of interim analyses/design adaptations [16](#_bookmark9)
   5. Risks and benefits [16](#_bookmark9)
      1. Blood sample volume [16](#_bookmark9)
5. Population [17](#_bookmark10)
   1. Inclusion criteria [17](#_bookmark10)
   2. Exclusion criteria [17](#_bookmark10)
6. Treatment [19](#_bookmark11)
   1. Study treatment [19](#_bookmark11)
      1. Investigational drug [19](#_bookmark11)
      2. Additional study treatments [19](#_bookmark11)
      3. Treatment arms/group [19](#_bookmark11)
   2. Other treatment(s) [19](#_bookmark11)
      1. Concomitant therapy [19](#_bookmark11)
      2. Prohibited medication after Screening [20](#_bookmark12)
      3. Restriction for study subjects [20](#_bookmark12)
   3. Subject numbering, treatment assignment, randomization [21](#_bookmark13)
      1. Subject numbering [21](#_bookmark13)
      2. Treatment assignment, randomization [21](#_bookmark13)
   4. Treatment blinding [22](#_bookmark14)
   5. Dose escalation and dose modification [22](#_bookmark14)
   6. Additional treatment guidance [22](#_bookmark14)
      1. Treatment compliance [22](#_bookmark14)
      2. Recommended treatment of adverse events [22](#_bookmark14)
      3. Emergency breaking of assigned treatment code [22](#_bookmark14)
   7. Preparation and dispensation [23](#_bookmark15)
7. Informed consent procedures [23](#_bookmark15)
8. Visit schedule and assessments [24](#_bookmark16)
   1. Screening [26](#_bookmark17)
      1. Information to be collected on screening failures [26](#_bookmark17)
   2. Subject demographics/other baseline characteristics [26](#_bookmark17)
   3. Efficacy [26](#_bookmark17)
   4. Safety [26](#_bookmark17)
      1. Laboratory evaluations [27](#_bookmark18)
      2. Electrocardiogram (ECG) [28](#_bookmark19)
      3. Pregnancy and assessments of fertility [28](#_bookmark19)
      4. Ophthalmic examination [28](#_bookmark19)
      5. Appropriateness of safety measurements [29](#_bookmark20)
   5. Additional assessments [29](#_bookmark20)
9. Study discontinuation and completion [30](#_bookmark21)
   1. Discontinuation [30](#_bookmark21)
      1. Discontinuation of study treatment [30](#_bookmark21)
      2. Withdrawal of informed consent [30](#_bookmark21)
      3. Lost to follow-up [31](#_bookmark22)
      4. Early study termination by the sponsor [31](#_bookmark22)
   2. Study completion and post-study treatment [31](#_bookmark22)
10. Safety monitoring and reporting [32](#_bookmark23)
    1. Definition of adverse events and reporting requirements [32](#_bookmark23)
       1. Adverse events [32](#_bookmark23)
       2. Serious adverse events [33](#_bookmark24)
       3. SAE reporting [34](#_bookmark25)
       4. Pregnancy reporting [35](#_bookmark26)
       5. Reporting of study treatment errors including misuse/abuse [35](#_bookmark26)
    2. Additional Safety Monitoring [36](#_bookmark27)
11. Data collection and database management [36](#_bookmark27)
    1. Data collection [36](#_bookmark27)
    2. Database management and quality control [37](#_bookmark28)
    3. Site monitoring [37](#_bookmark28)
12. Data analysis and statistical methods [38](#_bookmark29)
    1. Analysis sets [38](#_bookmark29)
    2. Subject demographics and other baseline characteristics [38](#_bookmark29)
    3. Treatments [38](#_bookmark29)
    4. Analysis of the primary endpoint(s) [38](#_bookmark29)
       1. Definition of primary endpoint(s) [38](#_bookmark29)
       2. Statistical model, hypothesis, and method of analysis [38](#_bookmark29)
       3. Handling of missing values/censoring/discontinuations [39](#_bookmark30)
       4. Sensitivity and Supportive analyses [39](#_bookmark30)
    5. Analysis of secondary endpoints [39](#_bookmark30)
       1. Safety endpoints [39](#_bookmark30)
    6. Interim analyses [39](#_bookmark30)
    7. Sample size calculation [39](#_bookmark30)
       1. Primary endpoint(s) [39](#_bookmark30)
13. Ethical considerations and administrative procedures [40](#_bookmark31)
    1. Regulatory and ethical compliance [40](#_bookmark31)
    2. Responsibilities of the investigator and IRB/IEC [40](#_bookmark31)
    3. Publication of study protocol and results [40](#_bookmark31)
    4. Quality Control and Quality Assurance [40](#_bookmark31)
14. Protocol adherence [41](#_bookmark32)
    1. Protocol amendments [41](#_bookmark32)
15. References [42](#_bookmark33)

## List of tables

Table 2-1 Objectives and endpoints [14](#_bookmark7)

Table 6-1 Investigational drug [19](#_bookmark11)

Table 6-2 Prohibited medication [20](#_bookmark12)

Table 6-3 Blinding and unblinding plan [22](#_bookmark14)

Table 8-1 Assessment Schedule [24](#_bookmark16)

Table 8-2 Assessments and Specifications [27](#_bookmark18)

Table 8-3 Laboratory Assessments [27](#_bookmark18)

Table 10-1 Guidance for capturing the study treatment errors including misuse/abuse [36](#_bookmark27)

## List of figures

Figure 3-1 Study Design [15](#_bookmark8)

## List of abbreviations

ADA anti-drug antibodies

AE adverse event

ALP alkaline phosphatase

ALT alanine aminotransferase

AMD age related macular degeneration

anti-VEGF antivascular endothelial growth factor AST aspartate aminotransferase

AV atrioventricular

BCVA best corrected visual acuity

bpm beats per minute

CFR code of federal regulations

CMO & PS Chief Medical Office and Patient Safety CNV choroidal neovascularization

CRA clinical research associate

CrCL creatinine clearance

CRF case report form

CRO clinical research organization

e.g. for example

ECG electrocardiogram

eCRF electronic case report form

EDC electronic data capture

EMA European Medicines Agency

EOS end of study

EU European Union

EudraCT European Clinical Trials Database FDA Food and Drug Administration

FU follow up

GCP Good Clinical Practice

GGT gamma-glutamyl transferase

h hour

HDL high-density lipoprotein

i.e. that is

ICF informed consent form

ICH International Conference on Harmonization of Technical Requirements for Registration of Pharmaceuticals for Human Use

IEC Independent Ethics Committee

IOP intraocular pressure

IRB Institutional Review Board

IVT intravitreal

kDa kilodalton

LAMA long-acting muscarinic antagonist

LDH low-density lipoproteins

LABA long-acting beta adrenergic

mAbs monoclonal antibodies

MDRD modification of diet in renal disease study MedDRA medical dictionary for regulatory activities μL microliter(s)

mg milligram(s)

mL milliliter(s)

mmHg millimeters of mercury

ms millisecond

OU both eyes

PMDA Pharmaceuticals and Medical Devices Agency q4w every 4 weeks

RBC red blood cell

RPE retinal pigment epithelium

SAE serious adverse event

SAP statistical analysis plan

scFv single-chain Fv

SE study eye

SGOT serum glutamic oxaloacetic transaminase

SGPT serum glutamic pyruvic transaminase

SOM study operations manual

SUSAR suspected unexpected serious adverse reaction US United States

WBC white blood cell

WHO World Health Organization WOCBP women of child bearing potential

## Glossary of terms

| Assessment | A procedure used to generate data required by the study |
| --- | --- |
| Biologic Samples | A biological specimen including, for example, blood (plasma, serum), saliva, tissue, urine, stool, etc. taken from a study subject |
| Dosage | Dose of the study treatment given to the subject in a time unit (e.g. 100 mg once a day, 75 mg twice a day) |
| Electronic Data Capture (EDC) | Electronic data capture (EDC) is the electronic acquisition of clinical study data using data collection systems, such as Web-based applications, interactive voice response systems and clinical laboratory interfaces. EDC includes the use of Electronic Case Report Forms (eCRFs) which are used to capture data transcribed from paper source forms used at the point of care |
| End of the clinical trial | The end of the clinical trial is defined as the last visit of the last subject or at a later point in time as defined by the protocol |
| Enrollment | Point/time of subject entry into the study at which informed consent must be obtained |
| Investigational drug/ treatment | The drug whose properties are being tested in the study |
| Medication number | A unique identifier on the label of medication kits |
| Other treatment | Treatment that may be needed/allowed during the conduct of the study (i.e. concomitant or rescue therapy) |
| Patient | An individual with the condition of interest for the study |
| Period | The subdivisions of the trial design (e.g. Screening, Treatment, Follow-up) which are described in the Protocol. Periods define the study phases and will be used in clinical trial database setup and eventually in analysis |
| Personal data | Subject information collected by the Investigator that is transferred to Novartis for the purpose of the clinical trial. This data includes subject identifier information, study information and biological samples. |
| Premature subject withdrawal | Point/time when the subject exits from the study prior to the planned completion of all study drug administration and/or assessments; at this time all study drug administration is discontinued and no further assessments are planned |
| Screen Failure | A subject who did not meet one or more criteria that were required for participation in the study |
| Study operational manual (SOM) | SOM provides additional operational guidance/details for study procedures and is not part of the Clinical Study Report |
| Source Data/Document | Source data refers to the initial record, document, or primary location from where data comes. The data source can be a database, a dataset, a spreadsheet or even hard-coded data, such as paper or eSource |
| Start of the clinical trial | The start of the clinical trial is defined as the signature of the informed consent by the first subject |
| Study treatment | Any single drug or combination of drugs or intervention administered to the subject as part of the required study procedures |
| Study treatment discontinuation | When the subject permanently stops taking any of the study drug(s) prior to the defined study treatment completion date (if any) for any reason; may or may not also be the point/time of study discontinuation |
| Subject | A trial participant |

| Subject number | A unique number assigned to each subject upon signing the informed consent. This number is the definitive, unique identifier for the subject and should be used to identify the subject throughout the study for all data collected, sample labels, etc. |
| --- | --- |
| Withdrawal of study consent (WoC) | Withdrawal of consent from the study occurs only when a subject does not want to participate in the study any longer and does not allow any further collection of personal data |

## Protocol summary

| **Protocol number** | CRTH258A2309 |
| --- | --- |
| **Full Title** | A Single-Arm, Open-Label, Multicenter, Phase IIIb Study to Collect Safety and Electrocardiogram Data on Brolucizumab 6 mg Intravitreal Treatment in Patients with Neovascular Age-Related Macular Degeneration |
| **Brief title** | Study to collect safety and ECG data on Brolucizumab 6 mg Intravitreal Treatment in Patients with wet AMD |
| **Sponsor and Clinical Phase** | Novartis/IIIb |
| **Investigation type** | Biologic |
| **Study type** | Interventional |
| **Purpose and rationale** | The purpose of this study is to collect ECG data after a single IVT injection of brolucizumab 6 mg in patients with neovascular age-related macular degeneration (nAMD). |
| **Primary Objective(s)** | The primary objective of this study is to collect information on ECG after intravitreal injection of brolucizumab 6 mg in patients with nAMD. |
| **Secondary Objectives** | The secondary objective is to collect safety data after intravitreal injection of brolucizumab 6 mg in patients with nAMD. |
| **Study design** | This is a single-arm, open-label, multicenter study to collect ECG data after a single IVT injection of brolucizumab 6 mg in patients with nAMD. Total study duration for each patient is up to 8 days. |
| **Population** | The study population will consist of male and female patients with nAMD. Approximately 10-15 patients ≥ 50 years old are expected to be enrolled in the US and Puerto Rico. |
| **Key Inclusion criteria** | - Signed informed consent MUST be obtained prior to participation in the study - Study eye is diagnosed with nAMD and deemed to be eligible for intravitreal injection at the discretion of the Investigator |

| **Key Exclusion criteria** | - Treatment with any ocular intravitreal injection in the study eye within the past 7 half-lives prior to Baseline - Diagnosis of ECG abnormalities including:   - Clinically significant cardiac arrhythmias, e.g., atrial fibrillation, sustained ventricular tachycardia, and clinically significant second or third degree AV block without a pacemaker   - Familial long QT syndrome or known family history of Torsades de Pointes   - Resting heart rate < 50 or > 90 bpm at screening   - Resting QTcF ≥ 450 ms (male) or ≥ 460 ms (female) at screening - Use of concomitant medications that are classified as known risk, conditional risk or possible risk to prolong QT/QTc interval within 7 half-lives of the drug prior to baseline - History of stroke (including transient ischemic attack, reversible ischemic neurological deficit, prolonged reversible ischemic neurological deficit) or myocardial infarction (ST or non-ST elevation myocardial infarction) at any time prior to baseline - Chronic kidney disease as determined as a CrCL at screening of < 60 ml/min/1.73 m2 as determined by the MDRD formula - Uncontrolled high blood pressure defined as a systolic value   ≥ 140 mmHg or diastolic value ≥ 90 mmHg at screening or baseline   - Systemic anti-VEGF therapy during the 6-month period prior to baseline - Electrolyte disturbances determined as out of normal range sodium, potassium or calcium serum concentrations at screening |
| --- | --- |
| **Study treatment** | RTH258 (brolucizumab) |
| **Efficacy assessments** | Not applicable |
| **Key safety assessments** | Electrocardiogram (ECG), Holter ECG Monitoring, Vital Signs, Ophthalmic Exam (including Intraocular Pressure, Biomicroscopy, and Ophthalmoscopy), Physical Exam, Screening Labs (Hematology, Clinical Chemistry, and Pregnancy) |
| **Data analysis** | There are no formal hypotheses in this study. Incidences between 20 and 24 h post-injection of clinically relevant treatment emergent changes in HR, PR, QRS, and QTc interval will be listed by patient. The frequency and proportion of patients with QTcF > 450, 480, and 500 ms between 20 and 24 h post-injection will be summarized. |
| **Key words** | neovascular age-related macular degeneration, AMD, nAMD, wet AMD, RTH258, brolucizumab, ECG, open-label |

# Introduction

## Background

Age-related macular degeneration (AMD) is a chronic eye disease characterized by progressive degenerative abnormalities in the central retina (macula) and is the leading cause of severe vision loss in people affecting 10%-13% of individuals over the age of 65 years in North America, Europe, and Australia ([Kawasaki et al 2010](#_bookmark33); [Rein et al 2009](#_bookmark33); [Smith et al 2001](#_bookmark33)). Genetic, environmental and health factors play an important role in the pathogenesis of the disease ([Wong et al 2014](#_bookmark33)).

AMD is classified into 2 clinical subtypes: the non-neovascular (atrophic) or dry form and the neovascular (exudative) or wet form ([Ferris et al 1984](#_bookmark33); [Lim et al 2012](#_bookmark33); [Miller 2013](#_bookmark33)). Neovascular AMD is characterized by the growth of abnormal new blood vessels (neovascularization) under the retinal pigment epithelium (RPE) or subretinal space from the subjacent choroid, termed choroidal neovascularization (CNV) ([Ferris et al 1984](#_bookmark33)). These newly formed vessels have an increased likelihood to leak blood and serum, damaging the retina by stimulating inflammation and scar tissue formation. This damage to the retina results in progressive, severe, and irreversible vision loss ([Shah and Del Priore 2007](#_bookmark33); [Shah and Del Priore 2009](#_bookmark33)). Without treatment, most affected eyes will have poor central vision (20/200) within 12 months ([Blinder et al 2003](#_bookmark33)).

VEGF has been shown to be elevated in patients with neovascular AMD, and is thought to play a key role in the neovascularization process ([Spilsbury et al 2000](#_bookmark33)). The use of intravitreal (IVT) pharmacotherapy targeting VEGF has significantly improved visual outcomes in patients with neovascular AMD ([Bloch et al 2012](#_bookmark33); [Campbell et al 2012](#_bookmark33)). Anti-VEGF treatments, such as ranibizumab (LUCENTIS®) and aflibercept (EYLEA®), inhibit VEGF signaling pathways and have been shown to halt the growth of neovascular lesions and resolve retinal edema.

**Brolucizumab**

Brolucizumab (RTH258, formerly ESBA1008) is a humanized single-chain Fv (scFv) antibody fragment inhibitor of vascular endothelial growth factor with a molecular weight of ~26 kDa. Brolucizumab is an inhibitor of VEGF-A and works by binding to the receptor binding site of the VEGF-A molecule, thereby preventing the interaction of VEGF-A with its receptors VEGFR1 and VEGFR2 on the surface of endothelial cells.

A low molecular weight (a surrogate for hydrodynamic radius) should increase drug distribution into the target site of action, ensuring effective control of anatomical disease activity. This could potentially translate into a more durable efficacy and a reduced treatment burden (number of injections) for the patient and health care professional.

**Brolucizumab in nAMD**

In a single ascending dose Phase I study (C-10-083), the median time until patients fulfilled protocol defined criteria for receipt of standard of care treatment was 30 days longer for brolucizumab 6 mg (P = 0.036) versus ranibizumab, with the maximum effect on best-corrected visual acuity (BCVA) and central subfield thickness (CSFT) reached at Week 6 for brolucizumab versus Week 4 for ranibizumab. In a separate repeat dosing Phase II study

(C-12-006, OSPREY) comparing brolucizumab 6 mg (n = 44) every 8 weeks (q8w), then every 12 weeks (q12w) administration, i.e. administration every q8w and q12w, respectively, against aflibercept (q8w administration, n = 45), brolucizumab achieved comparable visual outcome during the loading and q8w phase, with a lower number of patients requiring additional rescue treatments (5 out of 44 versus 10 out of 45, respectively). Brolucizumab demonstrated a trend for greater improvements and more stability in retinal anatomy during the 4 cycles of q8w dosing (up to Week 40).

In the Phase III Studies RTH258-C001 (HAWK) and RTH258-C002 (HARRIER),

brolucizumab demonstrated non-inferiority to aflibercept in mean change in BCVA from baseline to Week 48 in both trials using a non-inferiority margin (NIM) of 4 letters. In both studies, the treatment effect observed at Week 48 was largely maintained up to the end of the study (Week 96) in all treatment arms and results for BCVA-related secondary endpoints at Week 96 were overall comparable with those at Week 48. These results were achieved while a majority of patients on brolucizumab 6 mg – 56% in HAWK and 51% in HARRIER – were maintained on a q12w (every 12 weeks) dosing interval following the loading phase (baseline to Week 8) through Week 48. Patients in the brolucizumab 6 mg treatment group who were on q12w at Week 44 revealed probabilities of 81.5 % and 75.4% to remain on q12w up to week 92 in HAWK and HARRIER, respectively. Brolucizumab 6 mg achieved superior reductions in CSFT versus aflibercept in both the head-to-head and maintenance phases (P = 0.0016 and P = 0.0023 at Week 16 and Week 48, respectively, in HAWK; P < 0.0001 at both Week 16 and Week 48 in HARRIER). The greater reductions in CSFT observed at Week 48 with brolucizumab 6 mg or 3 mg compared to patients treated with aflibercept 2 mg were maintained through Week 96. Brolucizumab safety was comparable to aflibercept, with the overall incidence of adverse events (AEs) balanced across all treatment groups in both studies.

This study, CRTH258A2309, is an ECG collection study on request of the Pharmaceuticals and Medical Devices Agency (PMDA) of Japan. Patients from US and Puerto Rico, 50 years old or greater, diagnosed with nAMD will be eligible to participate. All enrolled patients will receive one (1) intravitreal dose of brolucizumab 6 mg at baseline and will be observed over a study period of 8 days with a follow up phone call on day 31.

## Purpose

The purpose of this study is to collect ECG data after a single IVT injection of brolucizumab 6 mg in patients with neovascular age-related macular degeneration (nAMD).

# Objectives and endpoints

**Table 2-1 Objectives and endpoints**

**Objective(s) Endpoint(s)**

**Primary Objective(s) Endpoint(s) for primary objective(s)**

- To collect information on ECG after intravitreal injection of brolucizumab 6 mg in patients with nAMD
- Incidence between 20 and 24 h post-injection of clinically relevant treatment emergent changes in HR, PR, QRS, and QTc (heart rate corrected QT using Fridericia’s formula,

QTcF) interval (ms)

**Secondary Objective(s) Endpoint(s) for secondary objective(s)**

- To collect safety data after intravitreal injection of brolucizumab 6 mg in patients with nAMD
- Any ocular and non-ocular AEs (including clinically relevant ECG abnormalities) until the end of the study

# Study design

This is a single-arm, open-label, multicenter study to collect ECG data after a single IVT injection of brolucizumab 6 mg in patients with nAMD.

All patients must provide informed consent before any study-specific procedure is performed. At the Screening and Baseline visits, patients are evaluated for study eligibility based on the inclusion/exclusion criteria. Patients who meet all the inclusion and none of the exclusion criteria will receive one single intravitreal injection (IVT) of brolucizumab 6 mg during the treatment phase of the study.

Triplicate 12-lead ECG recording will be performed at screening to determine eligibility. A second triplicate 12-lead ECG recording will be collected approximately 2h prior to the brolucizumab IVT injection on Day 1. Holter ECG recording will start approximately 1 h prior to the brolucizumab IVT injection and will end approximately 48h after the IVT injection. A third triplicate 12-lead ECG recording will be performed after the conclusion of Holter monitoring on Day 3.

The final study assessments will be performed at end of study (EOS) visit on Day 8, i.e. 1 week after injection. The EOS visit will be performed via a telephone call. Total study duration for each patient is up to 8 days. Additionally, a safety follow-up phone call will be performed on Day 31. Any AEs starting between Day 9 and Day 31 are collected in the source documents. All SAEs MUST be reported to Novartis safety according to instructions in [Section 10.1.3](#_bookmark25) SAE reporting.


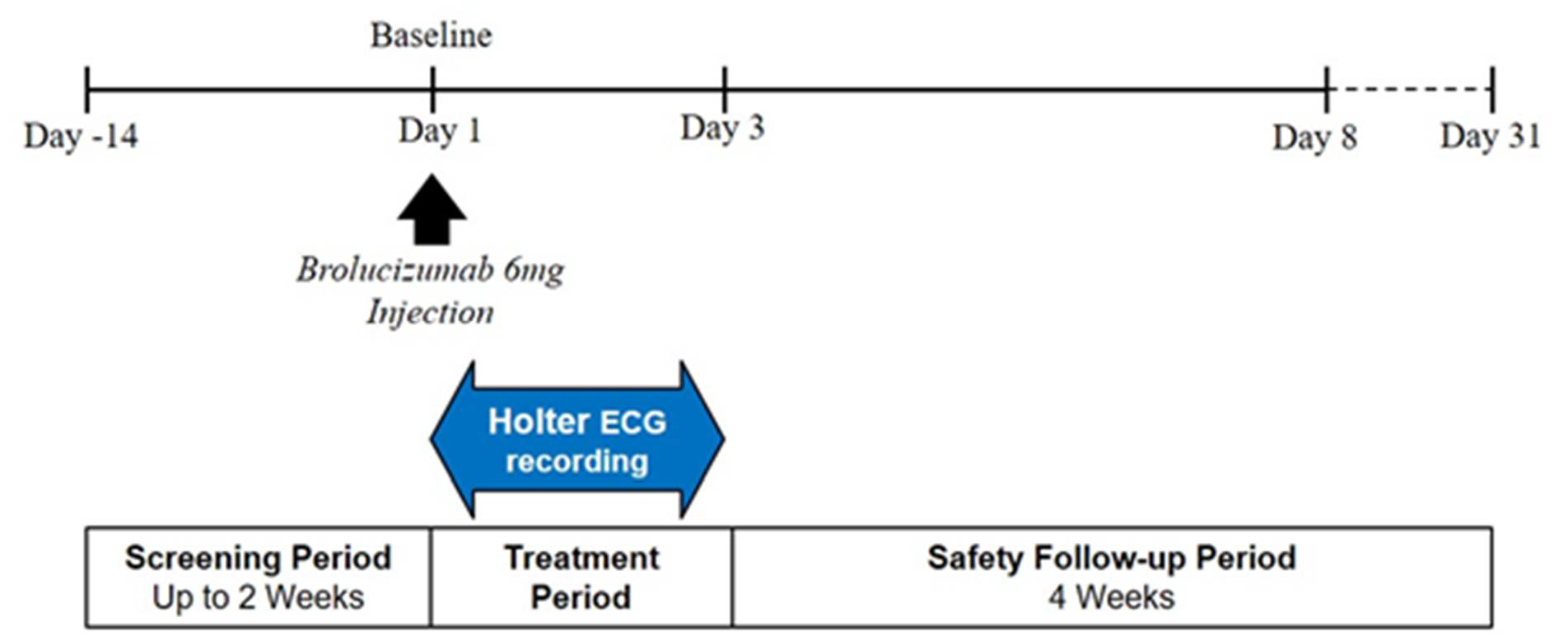
**Figure 3-1 Study Design**

# Rationale

## Rationale for study design

This multicenter study is designed to meet the request of the Pharmaceuticals and Medical Devices Agency (PMDA) of Japan to provide ECG data on approximately 10-15 patients at the time at which maximum systemic concentration is reached (Tmax) after a single injection of brolucizumab 6 mg. The study design, study duration, and patient population were assessed as adequate to meet the agency’s request. **The study is NOT a thorough QT study.** The patient population is described in [Section 5](#_bookmark10) Population below.

Furthermore, monoclonal antibodies (mAbs) are not expected to directly inhibit the function of ion channels responsible for cardiac repolarization; therefore, thorough QT studies are generally not required for biotherapeutics ([Malik et al 2010](#_bookmark33)). There are no known preclinical cardiovascular safety signals with brolucizumab. The systemic exposure was confirmed to be very low in human with a median AUCinf (Area under the concentration-time curve from time zero to infinity) of 5580 ng*h/mL (Investigator’s Brochure).

Approximately 1 h before a single injection of brolucizumab, a Holter monitor will provide continuous ECG monitoring for 48h; capturing the approximate time at which maximum systemic concentration is reached (median Tmax was approximately 24 hours post dose). A single injection is deemed sufficient because it was previously shown in the RTH258A2201 study that brolucizumab does not accumulate in the systemic circulation after repeated monthly injections (SHRIKE, RTH258E003).

Additionally long-term ECG data is being collected in an ongoing large Phase III study of brolucizumab 3 mg or 6 mg in Japanese patients with diabetic macular edema, CRTH258B2301. ECG data for the CRTH258B2301 study is collected at baseline and after 52 and 100 weeks of first treatment with brolucizumab 3 mg, 6 mg or aflibercept.

## Rationale for dose/regimen and duration of treatment

The dose and regimen for brolucizumab are based on the following considerations:

- Brolucizumab is well tolerated at a dose of 6 mg, based on the previous clinical Phase III program in which 1088 patients with nAMD received brolucizumab.
- Brolucizumab 6 mg is the maximum feasible dose for a 50 μL IVT injection based on brolucizumab physico-chemical properties and is the highest dose that has been tested. Brolucizumab 6 mg provides the higher systemic exposure after IVT injection as compared to the different dose strengths previously investigated in patients and is foreseen as the therapeutic dose. Thus, the proposed dose and route are appropriate to generate ECG data in its target population with brolucizumab.

## Rationale for choice of control drugs

Not applicable.

## Purpose and timing of interim analyses/design adaptations

Not applicable.

## Risks and benefits

Intravitreal brolucizumab 6 mg has been administered to 1355 patients with nAMD for up to 2 years treatment. Further details of the known and potential risks and benefits associated with brolucizumab are presented in the RTH258/brolucizumab Investigator’s Brochure.

All intravitreal injection procedures carry the risk of endophthalmitis, retinal detachment, vitreous or retinal hemorrhage, cataract, elevated intraocular pressure, and ocular inflammation. These conditions are apparent on eye exam and most cause symptoms that prompt the patient to seek immediate evaluation. With respect to endophthalmitis, current best clinical practices are used to reduce the risk of endophthalmitis. Despite these practices, endophthalmitis rates may approximate 1 in 1,000 to 1 in 20,000.

As with any antibody, brolucizumab may carry the risk of anaphylaxis or hypersensitivity reactions and may also elicit the formation of human anti-drug antibodies (ADA).

The risk to patients in this trial may be minimized by compliance with the eligibility criteria and study procedures, as well as close clinical monitoring.

### Blood sample volume

Approximately 6 mL of blood is planned to be collected over a period of up to 14 days (during Screening), from each patient as part of study screening. Additional samples may be required for safety monitoring.

The timing of blood sample collection is outlined in the Assessment Schedule ([Table 8-1](#_bookmark16))*.* A summary blood log is provided in the Site Operations Manual (SOM).

# Population

The study population will consist of male and female patients with nAMD. Approximately 10-15 patients ≥ 50 years old are expected to be enrolled in the US and Puerto Rico.

## Inclusion criteria

Patients eligible for inclusion in this study must meet **all** of the following criteria at screening:

1. Signed informed consent MUST be obtained prior to participation in the study
2. Study eye is diagnosed with nAMD and deemed to be eligible for intravitreal injection at the discretion of the Investigator

## Exclusion criteria

Patients meeting any of the following criteria are **NOT** eligible for inclusion in this study. No additional exclusions may be applied by the investigator, in order to ensure that the study population will be representative of all eligible patients.

1. Concomitant conditions or ocular disorders in the study eye at screening which may cause safety concerns on the judgement of the investigator
2. Any active intraocular or periocular or systemic infection or active intraocular inflammation at Baseline
3. Treatment with any ocular intravitreal injection in the study eye within the past 7 half-lives prior to Baseline
4. Intraocular surgery, prior long-acting therapeutic agent, or ocular drug release device implantation (approved or investigational) in the study eye any time during the past 3 months prior to Baseline
5. Diagnosis of ECG abnormalities including:
   - Clinically significant cardiac arrhythmias, e.g., atrial fibrillation, sustained ventricular tachycardia, and clinically significant second or third degree AV block without a pacemaker
   - Familial long QT syndrome or known family history of Torsades de Pointes
   - Resting heart rate < 50 or > 90 bpm at screening
   - Resting QTcF ≥ 450 ms (male) or ≥ 460 ms (female) at screening
6. Use of concomitant medications that are classified as known risk, conditional risk or possible risk to prolong QT/QTc interval within 7 half-lives prior to Baseline
7. History of stroke (including transient ischemic attack, reversible ischemic neurological deficit, prolonged reversible ischemic neurological deficit) or myocardial infarction (ST or non-ST elevation myocardial infarction) at any time prior to baseline
8. Chronic kidney disease as determined as a CrCL at screening of < 60 ml/min/1.73 m2 as determined by the MDRD formula
9. Uncontrolled high blood pressure defined as a systolic value ≥ 140 mmHg or diastolic value ≥ 90 mmHg at screening or baseline
10. Systemic anti-VEGF therapy during the 6-month period prior to baseline
11. Electrolyte disturbances determined as out of normal range sodium, potassium or calcium serum concentrations at screening
12. Concomitant intake of long-acting muscarinic antagonist (LAMA)/ long-acting beta adrenergic (LABA) combination therapy
13. History of hypersensitivity to any of the study drugs or its excipients or to drugs of similar classes as assessed by the investigator
14. Use of systemic or ocular (including intravitreal) investigational drugs within 7 half-lives of baseline, (or within 30 days/until the expected pharmacodynamic effect has returned to baseline), whichever is longer or longer if required by local regulations (observational clinical studies solely involving over-the-counter vitamins, supplements, or diets are not exclusionary)
15. Pregnant or nursing (lactating) women

Women of child-bearing potential, defined as all women physiologically capable of becoming pregnant, **unless** they are using highly effective methods of contraception while taking study treatment. Highly effective contraception methods include:

- Total abstinence (when this is in line with the preferred and usual lifestyle of the patient. Periodic abstinence (e.g. calendar, ovulation, symptothermal, post-ovulation methods) and withdrawal are not acceptable methods of contraception
- Female sterilization (have had surgical bilateral oophorectomy with or without hysterectomy), total hysterectomy, or tubal ligation at least six weeks before taking study treatment. In case of oophorectomy alone, only when the reproductive status of the woman has been confirmed by follow up hormone level assessment
- Male sterilization (at least 6 months prior to screening); For female patients on the study, the vasectomized male partner should be the sole partner for that patient
- Use of oral (estrogen and progesterone), injected, or implanted hormonal methods of contraception or placement of an intrauterine device (IUD) or intrauterine system (IUS), or other forms of hormonal contraception that have comparable efficacy (failure rate <1%), for example, hormone vaginal ring or transdermal hormone contraception

In the case of use of oral contraception, women must be stable on the same pill for a minimum of 3 months before taking study treatment.

Women are considered post-menopausal and not of child bearing potential if they have had 12 months of natural (spontaneous) amenorrhea with an appropriate clinical profile (e.g. age appropriate history of vasomotor symptoms) or have had surgical bilateral oophorectomy (with or without hysterectomy), total hysterectomy, or tubal ligation at least six weeks ago. In the case of oophorectomy alone, only when the reproductive status of the woman has been confirmed by follow up hormone level assessment is she considered not of child-bearing potential.

If local regulations deviate from the contraception methods listed above to prevent pregnancy, local regulations apply and is described in the informed consent form (ICF).

# Treatment

## Study treatment

Investigational product (IP) must be stored at 2° to 8°C (35.6° to 46.4°F); DO NOT FREEZE; protect from light.

Details on the requirements for storage and management of study treatment, and instructions for patient numbering, prescribing/dispensing and taking study treatment are outlined in the SOM.

### Investigational drug

**Table 6-1 Investigational drug**

**Investigational Drug (Name and Strength)**

**Pharmaceutical Dosage Form**

**Route of Administration**

**Supply Type Sponsor (global or**

**local)**

Brolucizumab (RTH258)

6 mg / 0.05 mL

Solution for injection Intravitreal

Injection

Glass Vials (in open label patient kit)

Novartis (global)

Brolucizumab (RTH258) will be provided to the study sites in a single use, sterile glass vial containing sufficient brolucizumab to deliver a 6 mg dose when administering a volume of 0.05 mL. The content of the study drug vials must not be split (single use vials). Novartis will provide sufficient supplies of brolucizumab for treatment use to allow for completion of the study.

### Additional study treatments

No other investigational treatment beyond the single injection of brolucizumab 6 mg is included in this trial.

### Treatment arms/group

All eligible patients enrolled in this trial will receive a single injection of brolucizumab 6 mg at the Baseline visit (Day 1).

## Other treatment(s)

Treatment after the completion of the study follows current standard of care or PI discretion. Standard of care includes non-investigational therapies that are used according to clinical practice for the treatment of nAMD in the US or Puerto Rico. Other treatments, including off-label use and other clinical interventional trials or managed access programs, are prescribed or recommended under the responsibility of the investigator.

### Concomitant therapy

All medications, procedures and significant non-drug therapies (including physical therapy and blood transfusions) administered after the patient was enrolled into the study must be recorded on the appropriate Case Report Forms (CRFs).

Each concomitant drug must be individually assessed against all exclusion criteria/prohibited medication. If in doubt the investigator should contact the Novartis medical monitor before randomizing a patient or allowing a new medication to be started. If the patient is already enrolled, the investigator must contact Novartis to determine if the patient should continue participation in the study.

### Prohibited medication after Screening

Use of the treatments displayed in the below table are **NOT allowed after screening**.

**Table 6-2 Prohibited medication**

**Medication Prohibition period Action taken**

QT prolonging agent* From Screening to Day 8 (EOS) visit

Study discontinuation

Anti-VEGF therapy other than assigned study medication in both eyes†

From Screening to Day 8 (EOS) visit

Study discontinuation

Systemic Anti-VEGF therapy From Screening to Day 8 (EOS) visit

Study discontinuation

Intraocular or periocular injections of corticosteroids (except if treatment for AE) in both eyes

From Screening to Day 8 (EOS) visit

Study discontinuation

*Please refer to https://crediblemeds.org/pdftemp/pdf/CombinedList.pdf

†During the study, if the fellow eye develops visual impairment, it may also be treated with standard of care (i.e. treatment of the fellow eye with anti-VEGF medication other than brolucizumab) at the discretion of the investigator. If the fellow eye treatment is required before Day 8, then the patient will be early discontinued from the trial.

### Restriction for study subjects

For the duration of the study, patients should be informed and reminded of the restrictions outlined in this section. The below advices are suggested guidelines.

**Nonconformity to these advices will NOT result in a protocol deviation.**

- - - 1. **Dietary restrictions and smoking**

1. No alcohol for 24 hours before dosing until after the study evaluation on Day 3.
2. No cannabis or cannabidiol oil containing products for 24 hours before dosing until after the study evaluation on Day 3.
3. Intake of xanthine (e.g. caffeine) containing food or beverages (i.e., coffee, tea, soda, chocolate) must be limited to 4 cups/day or less 24 hours prior to dosing until after the study evaluation on Day 3.
4. Patients should maintain adequate hydration 24 hours prior to dosing until after the study evaluation on Day 3.
   - - 1. **Other restrictions**

During Holter Monitoring:

1. No strenuous physical exercise (e.g. weight training, aerobics, football, etc.) 24 hours prior to the treatment visit and for the entire duration you are wearing the Holter Monitor (until removal on Day 3). At approximately 22-24 hours after the IVT injection, try to avoid activity by laying down and resting for 1-2 hours.
2. No bathing or swimming from the time the Holter Monitor electrodes are placed on Day 1 until it is removed on Day 3.
3. Try to avoid metal detectors, magnets, microwave ovens, electric blankets, electric razors and electric toothbrushes while wearing the Holter monitor.
4. Try to keep cellphones, portable music players, and other devices that may exert an electric frequency at least 6 inches from the Holter monitor.

## Subject numbering, treatment assignment, randomization

### Subject numbering

The patient number assigned to a patient at screening remains the unique identifier for the patient throughout the study. For information on patient numbering, please see ‘Subject numbering’ section in the Site Operations Manual.

### Treatment assignment, randomization

Treatment assignment and randomization are not applicable. This is a single-arm, open-label study and all eligible patients will receive the investigational treatment.

## Treatment blinding

Treatment blinding is not applicable because this is a single arm, open-label study. All patients, trial site, and Sponsor staff will know the treatment provided to the patient.

**Table 6-3 Blinding and unblinding plan**

|  | **Time or Event** | | | |
| --- | --- | --- | --- | --- |
| **Role** | **Randomization list generated** | **Treatment allocation & dosing** | **Safety event (single patient unblinded)** | **Interim Analysis & dose escalation** |
| Patients | N/A | UI | UI | N/A |
| Site staff | N/A | UI | UI | N/A |
| Global Clinical Supply and Randomization Office | N/A | UI | UI | N/A |
| Statistician/statistical programmer/ data analysts | N/A | UI | UI | N/A |
| All other sponsor staff not identified above (trial team, project team, management & decision boards, support functions) | N/A | UI | UI | N/A |

Key:

UI: Allowed to be unblinded on individual patient level B: Remains blinded

NA: Not applicable to this study

## Dose escalation and dose modification

Investigational or other study treatment dose adjustments are not permitted.

## Additional treatment guidance

### Treatment compliance

The patient will receive one intravitreal injection of brolucizumab 6 mg performed by a qualified ophthalmologist. The injection must be recorded on the appropriate eCRF page.

### Recommended treatment of adverse events

Medication used to treat adverse events (AEs) must be recorded on the appropriate CRF.

### Emergency breaking of assigned treatment code

Emergency breaking of the assigned treatment code is not applicable because this is a single arm, open label study.

## Preparation and dispensation

Each study site will be supplied with study drug in packaging as described under the investigational drugs section. Drug preparation instructions are included in the SOM.

# Informed consent procedures

Eligible patients may only be included in the study after providing (witnessed, where required by law or regulation), IRB/IEC-approved informed consent.

If applicable, in cases where the patient's representative(s) gives consent (if allowed according to local requirements), the patient must be informed about the study to the extent possible given his/her understanding. If the patient is capable of doing so, he/she must indicate agreement by personally signing and dating the written informed consent document.

Informed consent must be obtained before conducting any study-specific procedures (e.g. all of the procedures described in the protocol). The process of obtaining informed consent must be documented in the patient source documents.

Novartis will provide the investigators a separate document with a proposed informed consent form that complies with the ICH/GCP guidelines and regulatory requirements and is considered appropriate for this study. Any changes to the proposed consent form suggested by the investigator must be agreed by Novartis before submission to the IRB/IEC.

Information about common side effects already known about the investigational drug can be found in the IB. This information will be included in the patient informed consent and should be discussed with the patient during the study as needed. Any new information regarding the safety profile of the investigational drug that is identified between IB updates will be communicated as appropriate, for example, via an investigator notification or an aggregate safety finding. New information might require an update to the informed consent and then must be discussed with the patient.

Women of child bearing potential must be informed that taking the study treatment may involve unknown risks to the fetus if pregnancy were to occur during the study and agree that in order to participate in the study they must adhere to the contraception requirements.

A copy of the approved version of all consent forms must be provided to Novartis after IRB/IEC approval.

Refer to the Site Operations Manual for a complete list of ICFs included in this study.

# Visit schedule and assessments

The Assessment Schedule ([Table 8-1](#_bookmark16)) lists all of the assessments and when they are performed. All data obtained from these assessments must be supported in the patient’s source documentation.

Patients should be seen for all visits/assessments as outlined in the Assessment Schedule ([Table 8-1](#_bookmark16)) or as close to the designated day/time as possible. Missed or rescheduled visits will not lead to automatic discontinuation. Patients who prematurely discontinue the study for any reason should be scheduled for a visit as soon as possible, at which time all of the assessments listed for the final visit will be performed. At this final visit, the adverse event and concomitant medications recorded on the CRF.

Additional details regarding the visit schedule and assessments are located in the SOM.

**Table 8-1 Assessment Schedule**

| **Period** | **Screening** | **Treatment** | | **Safety Follow-up (FU)** | |
| --- | --- | --- | --- | --- | --- |
| **Visit Name** | **Screening** | **Day 1 (Baseline)** | **Day 3 (Visit 2)** | **End of Study (EOS)** | **Safety Follow-up (FU)** |
| **Days** | **-14 to -1** | **1** | **3** | **8** | **31** |
| **Visit Number** | **1** | **101** | **102** | **199** | **N/A** |
| Informed consent | X |  |  |  |  |
| Inclusion / exclusion criteria | S | S |  |  |  |
| Demography | X |  |  |  |  |
| Medical history/current medical conditions | X |  |  |  |  |
| Prior/concomitant medications | X | X | X | X |  |
| Physical examination | S |  |  |  |  |
| Vital signs (Blood Pressure and Pulse Rate) | X | X2 | X3 |  |  |
| Electrocardiogram (ECG) | X | X2 | X |  |  |
| Holter ECG Monitoring |  | X4 | X5 |  |  |
| Hematology | X |  |  |  |  |
| Clinical chemistry | X |  |  |  |  |
| Pregnancy and assessments of fertility1 | X |  |  |  |  |

| **Period** | **Screening** | **Treatment** | | **Safety Follow-up (FU)** | |
| --- | --- | --- | --- | --- | --- |
| **Visit Name** | **Screening** | **Day 1 (Baseline)** | **Day 3 (Visit 2)** | **End of Study (EOS)** | **Safety Follow-up (FU)** |
| **Days** | **-14 to -1** | **1** | **3** | **8** | **31** |
| **Visit Number** | **1** | **101** | **102** | **199** | **N/A** |
| Intraocular Pressure (IOP) | OU | SE6 |  |  |  |
| Ophthalmic Exam | OU | SE6 |  |  |  |
| Adverse Events | X | X | X | X | S |
| Telephone follow-up |  |  |  | X | S |
| Study drug administration |  | X |  |  |  |
| Study completion information |  |  |  | X |  |

X = assessment to be recorded in the clinical database or received electronically from a vendor OU = Both Eyes

S = assessment to be recorded in the source documentation only SE = Study Eye

1 Women of childbearing potential only. Urine pregnancy tests will be performed unless local regulations require a serum pregnancy test.

2 Pre-Injection Procedure, performed before Holter monitoring start

3 Remove Holter Monitor before Performing Procedure(s)

4 Holter monitoring will start approximately 1 h pre dose

5 Holter monitoring will end approximately 48 h post dose

6 Assessment performed pre-dose at Baseline and performed post-dose, if required, at Baseline. Post-dose clinically significant abnormal findings should be recorded as an AE.

## Screening

Patients will be screened for eligibility for up to 14 days prior to the Baseline visit. It is permissible to re-screen a patient if s/he fails the initial screening; however, each must be discussed and agreed with by the Sponsor on a case-by-case basis. Information on what data must be collected for screening failures and further information on re-screening is outlined in the Site Operations Manual.

In the case where a safety laboratory assessment at screening indicates an electrolyte imbalance, the investigator should investigate if the electrolyte disturbance is due to a chronic underlying disease or if is the sequela of an intercurrent illness (e.g. such as vomiting or diarrhea due to a recent stomach virus or flu). If the electrolyte imbalance is due to a chronic underlying disease, then the patient must be excluded. If the electrolyte imbalance is due to an intercurrent illness and the investigator believes the intercurrent illness has adequately resolved, then the investigator may repeat the test once prior to treatment. If the repeat value remains outside of the specified ranges, the patient must be excluded from the study.

### Information to be collected on screening failures

Patients who sign an informed consent form and who are subsequently found to be ineligible, prior to receiving the injection, will be considered a screen failure. Information on what data must be collected for screening failures is outlined in the Site Operations Manual.

## Subject demographics/other baseline characteristics

Patient demographic and baseline characteristic data are to be collected on all patients. Relevant medical history/current medical condition present before signing the informed consent will be recorded. Investigators will have the discretion to record abnormal test findings on the appropriate CRF whenever, in their judgment, the test abnormality occurred prior to the informed consent signature. Details are outlined in the Site Operations Manual.

## Efficacy

Efficacy / Pharmacodynamics are not measured in this study.

## Safety

Safety assessments are specified below with the assessment schedule detailing when each assessment is to be performed. For details on adverse event (AE) collection and reporting, refer to the AE section ([Section 10.1.1](#_bookmark23)). The methods, assessment, specification, and recording for each assessment will be detailed in the SOM.

**Table 8-2 Assessments and Specifications**

**Assessment Specification**

Physical examination

A physical examination will be performed at Screening, as a general health check according to local clinical practice.

Clinically relevant findings that are present prior to signing informed consent must be included in the eCRF capturing Medical History. Significant findings identified after providing written informed consent which meet the definition of an Adverse Event must be recorded on the appropriate AE eCRF page.

Vital signs Vital signs include BP (systolic and diastolic pressure in mmHg) and pulse rate (beats per minute) measurements (see SOM Section 3.2 Vital signs for details). In case there is an elevated blood pressure measurement as specified in the

exclusion criteria, at the screening and baseline visits, the blood pressure

measurement may be repeated up to two times within 30 minutes of the first measurement (i.e. 3 measurements maximum within 30 min of the first measurement). If the final repeat measurement is elevated, then the patient is not eligible to be enrolled into the study.

If the case there is an out of range heart rate at screening and baseline, two additional repeat readings can be obtained, so that a total of up to three consecutive assessments are made, with the subject seated quietly for approximately five minutes preceding each repeat assessment. The last reading must be within the ranges provided in the eligibility criteria in order for the subject to qualify.

On Day 1, vital signs will be measured **before** the administration of study medication. The results are recorded in the eCRF.

Ophthalmic Exam Intraocular Pressure (IOP), Biomicroscopy (slit lamp examination), Ophthalmoscopy (funduscopy) will be performed (see protocol [Section 8.4.4](#_bookmark19) for details).

### Laboratory evaluations

Local laboratories will be used for analysis of all specimens collected at screening that will be used to evaluate systemic status. Any additional samples may be taken for safety monitoring. Collection of these samples will be performed according to the local clinical practice. Clinically significant abnormalities must be recorded as either medical history/current medical conditions or adverse events as appropriate.

**Table 8-3 Laboratory Assessments**

**Test Category Test Name**

Hematology Hematocrit, hemoglobin, red blood cell (RBC) count, white blood cell (WBC) count with differential (absolute and percentage of neutrophils, lymphocytes, monocytes, eosinophils, and basophils), and quantitative platelet count

Chemistry Serum electrolytes (sodium, potassium, chloride, phosphorus, calcium, magnesium), uric acid, urea nitrogen, creatinine, albumin, glucose, total protein, total bilirubin and direct bilirubin, serum glutamic oxaloacetic transaminase (SGOT)/ aspartate aminotransferase (AST), serum glutamic pyruvic transaminase (SGPT)/ alanine aminotransferase (ALT), gamma-glutamyl transferase (GGT), alkaline phosphatase (ALP), lactate dehydrogenase (LDH), troponin, high-density lipoprotein (HDL), low-density lipoprotein (LDL)

Pregnancy Test Urine pregnancy test for women of childbearing potential. If required by local regulations, serum testing is accepted

### Electrocardiogram (ECG)

Full details of all procedures relating to the ECG collection and reporting are contained in the core reading center technical manual and in the Site Operations Manual*.*

Triplicate 12 lead ECGs (performed at Screening, Baseline pre-injection, and Day 3) and 12 lead Holter ECGs will be collected with ECG machines supplied by the core ECG reading center. All ECGs will be sent to the central ECG reading center. Central ECG reading will be used to determine eligibility at Screening.

A monitoring or review process should be in place for clinically significant ECG findings throughout the study. Only clinically significant abnormalities must be reported as adverse events.

The Holter ECG recording is performed to capture comprehensive ECG data and should start approximately one hour before IVT injection. The recording will end approximately 48 h after injection.

In the event that a clinically significant ECG abnormality is identified at the site (e.g. severe arrhythmia, conduction abnormality of QTcF > 500 ms), a copy of the assessment is sent to the core laboratory for expedited review if applicable, and the ECG is repeated to confirm the diagnosis. If the patient is hemodynamically compromised, the investigator or a medically qualified person must initiate appropriate safety procedures without delay (for example cardioversion).

Clinically significant abnormalities must be recorded on the eCRF as either medical history/current medical conditions or adverse events as appropriate.

### Pregnancy and assessments of fertility

All pre-menopausal women who are not surgically sterile will have pregnancy testing at Screening. Additional pregnancy testing might be performed if requested by local requirements.

Local pregnancy testing and associated results will not be collected on the eCRF.

Highly effective contraception is required for women of child-bearing potential during the study drug administration and for 3 months after the investigational medication is administered.

### Ophthalmic examination

The ophthalmic examination (including IOP, Biomicroscopy, and Ophthalmoscopy) will be performed at Screening and Baseline (pre-dose and post-dose) as noted below:

- **Intraocular pressure (IOP)** – IOP will be measured in mmHg and entered in the eCRF.
  - Screening: IOP is assessed in both eyes (OU) at Screening.
  - Baseline: IOP is assessed in the study eye (SE) ONLY; pre-dose and post-dose measurements are required at Baseline.
    - Post-dose IOP should be assessed, approximately 30 - 60 minutes after the IVT injection and, if ≥ 25 mmHg, the assessment should be repeated until back to normal.
    - Treatment and close monitoring of an elevated IOP should be performed by the investigator according to clinical practice
- **Biomicroscopy (slit lamp examination)** – The slit lamp examination will be completed to examine the structures of the study eye. The results of the examination must be recorded in the source documents.
  - Screening: Biomicroscopy is assessed in both eyes (OU).
  - Baseline: Biomicroscopy is assessed in the study eye (SE) ONLY; pre-dose and, if required, post dose examinations.
- **Ophthalmoscopy (funduscopy)** – Ophthalmoscopy will be completed to examine the peripheral retina and confirm that an intravitreal injection can be safely performed. The results of the examination must be recorded in the source documents.
  - Screening: Ophthalmoscopy is assessed in both eyes (OU) at Screening.
  - Baseline: Ophthalmoscopy is assessed in the study eye (SE) ONLY; pre-dose and, if required, post-dose examinations

Note: Pupil dilation for the slit lamp examination and ophthalmoscopy is optional according to local practice.

Clinically significant abnormal findings (as judged by the masked investigator) from slit lamp or ophthalmoscopy observations should be recorded as an AE in the eCRF.

### Appropriateness of safety measurements

Standard ECG intervals and morphology variables will be assessed, and QTcF and QTcB will be calculated. Cardiac assessments will be based on the QTcF, although QTcB will be used for comparative and historical control. ECG data is collected at several time points (Screening, Baseline and then continuously between Baseline 1h pre-injection through 48h, and on Day 3). It is appropriate to explore the within- and between-patient exposure-effect relationship.

Laboratory samples will be drawn at Screening in order to identify a potential electrolyte imbalance (e.g. hypokalemia) that might affect ECG parameters and morphology.

The other safety assessments selected are standard for this indication/patient population.

## Additional assessments

No additional tests will be performed on patients entered into this study.

# Study discontinuation and completion

## Discontinuation

### Discontinuation of study treatment

Since this is a single dose study, discontinuation of study treatment is not applicable.

Patients who decide they do not wish to participate in the study further after the single dose should NOT be considered withdrawn from the study UNLESS they withdraw their consent (see [Section 9.1.2](#_bookmark21) Withdrawal of informed consent). **Where possible, they should return for the assessments indicated** in the Assessment Schedule ([Table 8-1](#_bookmark16)). If they fail to return for these assessments for unknown reasons, every effort (e.g. telephone, e-mail, letter) should be made to contact the patient/pre-designated contact as specified in the lost to follow-up, see [Section 9.1.3](#_bookmark22) Lost to follow-up. This contact should preferably be done according to the study visit schedule.

If the patient cannot or is unwilling to attend any visit(s), the site staff should maintain regular telephone contact with the patient, or with a person pre-designated by the patient. This telephone contact should preferably be done according to the study visit schedule.

- - - 1. **Replacement policy**

Patients will not be replaced on study. However, if a patient is considered as non-evaluable, enrollment of a new patient will be considered by Novartis if there are less than the required number of patients that complete the study.

### Withdrawal of informed consent

Patients may voluntarily withdraw consent to participate in the study for any reason at any time. Withdrawal of consent occurs only when a patient:

- Does not want to participate in the study anymore, and
- Does not allow further collection of personal data

In this situation, the investigator should make a reasonable effort (e.g. telephone, e-mail, letter) to understand the primary reason for the patient’s decision to withdraw his/her consent and record this information.

Study treatment must not be given (if not yet injected) and no further assessments conducted, and the data that would have been collected at subsequent visits will be considered missing.

Further attempts to contact the patient are not allowed unless safety findings require communicating or follow-up.

All efforts should be made to complete the assessments prior to study withdrawal. A final evaluation at the time of the patient’s study withdrawal should be made as detailed in the Assessment Schedule ([Table 8-1](#_bookmark16)).

Novartis will continue to keep and use collected study information (including any data resulting from the analysis of a patient’s samples until the time of withdrawal) according to applicable law.

All biological samples not yet analyzed at the time of withdrawal may still be used for further testing/analysis in accordance with the terms of this protocol and of the informed consent form.

### Lost to follow-up

For patients whose status is unclear because they fail to appear for study visits without stating an intention to discontinue or withdraw, the investigator must show "due diligence" by documenting in the source documents steps taken to contact the patient, e.g. dates of telephone calls, registered letters, etc. A patient should not be considered as lost to follow-up until due diligence has been completed or until the end of the study*.*

### Early study termination by the sponsor

The study can be terminated by Novartis at any time. Reasons for early termination:

- Unexpected, significant, or unacceptable safety risk to patients enrolled in the study
- Decision based on recommendations from applicable board(s) after review of safety and efficacy data
- Discontinuation of study drug development

In taking the decision to terminate, Novartis will always consider patient welfare and safety. Should early termination be necessary, patients must be seen as soon as possible and treated as a prematurely withdrawn patient. The investigator may be informed of additional procedures to be followed in order to ensure that adequate consideration is given to the protection of the patient’s interests. The investigator or sponsor depending on local regulation will be responsible for informing IRBs/IECs of the early termination of the trial.

## Study completion and post-study treatment

Study completion is defined as when the last patient finishes their End of Study (EOS) visit and any repeat assessments associated with this visit have been documented and followed-up appropriately by the Investigator, or, in the event of an early study termination decision, the date of that decision*.*

All treated patients will have a safety follow-up call conducted on Day 31. The information collected during this call is kept as source documentation. All SAEs reported during this time period must be reported as described in [Section 10.1.3](#_bookmark25) SAE reporting and SOM. Documentation of attempts to contact the patient are recorded in the source documentation*.*

Continuing care should be provided by the investigator and/or referring physician after the conclusion of the trial based on patient availability for follow-up.

This care may include:

- Enrollment in an extension study or managed access program, if any are available
- Initiation of standard of care treatment (approved treatment for nAMD) or other treatment, under the responsibility of the investigator and/or referring physician

# Safety monitoring and reporting

## Definition of adverse events and reporting requirements

### Adverse events

An adverse event (AE) is any untoward medical occurrence (e.g., any unfavorable and unintended sign [including abnormal laboratory findings], symptom or disease) in a patient or clinical investigation patient after providing written informed consent for participation in the study. Therefore, an AE may or may not be temporally or causally associated with the use of a medicinal (investigational) product.

The investigator has the responsibility for managing the safety of the individual patient and identifying adverse events.

Novartis qualified medical personnel will be readily available to advise on trial related medical questions or problems.

The occurrence of adverse events must be sought by non-directive questioning of the patient at each visit during the study. Adverse events also may be detected when they are volunteered by the patient during or between visits or through physical examination findings, laboratory test findings, or other assessments.

Adverse events must be recorded under the signs, symptoms, or diagnosis associated with them, accompanied by the following information (as far as possible) (if the event is serious refer to [Section 10.1.2](#_bookmark24) Serious adverse events):

1. The severity grade:
   - mild: usually transient in nature and generally not interfering with normal activities
   - moderate: sufficiently discomforting to interfere with normal activities
   - severe: prevents normal activities
2. Its relationship to the study treatment*.* If the event is due to lack of efficacy or progression of underlying illness (i.e. progression of the study indication) the assessment of causality will usually be ‘Not suspected.’ The rationale for this guidance is that the symptoms of a lack of efficacy or progression of underlying illness are not caused by the trial drug, they happen in spite of its administration and/or both lack of efficacy and progression of underlying disease can only be evaluated meaningfully by an analysis of cohorts, not on a single patient
3. Its duration (start and end dates) or if the event is ongoing, an outcome of not recovered/not resolved must be reported
4. Whether it constitutes an SAE (see [Section 10.1.2](#_bookmark24) Serious adverse events for definition of SAE) and which seriousness criteria have been met
5. Action taken regarding with study treatment

All adverse events must be treated appropriately. Treatment may include one or more of the following:

- - Dose not changed
  - Dose reduced/increased
  - Drug interrupted/withdrawn

1. Its outcome

Conditions that were already present at the time of informed consent should be recorded in medical history of the patient.

Adverse events (including lab abnormalities that constitute AEs) should be described using a diagnosis whenever possible, rather than individual underlying signs and symptoms.

Adverse event monitoring should be continued for at least 30 days following the last dose of study treatment.

Once an adverse event is detected, it must be followed until its resolution or until it is judged to be permanent (e.g. continuing at the end of the study), and assessment must be made at each visit (or more frequently, if necessary) of any changes in severity, the suspected relationship to the interventions required to treat it, and the outcome.

Information about adverse drug reactions for the investigational drug can be found in the Investigator’s Brochure (IB).

Abnormal laboratory values or test results constitute adverse events only if they fulfill at least one of the following criteria:

- - they induce clinical signs or symptoms
  - they are considered clinically significant
  - they require therapy

Clinically significant abnormal laboratory values or test results must be identified through a review of values outside of normal ranges/clinically notable ranges, significant changes from baseline or the previous visit, or values which are considered to be non-typical in patients with the underlying disease.

Follow the instructions found in the Site Operations Manual for data capture methodology regarding AE collection for patients that fail screening.

### Serious adverse events

An SAE is defined as any adverse event appearance of (or worsening of any pre-existing) undesirable sign(s), symptom(s) or medical conditions(s)) which meets any one of the following criteria:

- Fatal
- Life-threatening

Life-threatening in the context of a SAE refers to a reaction in which the patient was at risk of death at the time of the reaction; it does not refer to a reaction that hypothetically might have caused death if it were more severe (please refer to the [ICH-E2D Guidelines 2004](#_bookmark33)).

- Results in persistent or significant disability/incapacity
- Constitutes a congenital anomaly/birth defect
- Requires inpatient hospitalization or prolongation of existing hospitalization, unless hospitalization is for:
  - Routine treatment or monitoring of the studied indication, not associated with any deterioration in condition
  - Elective or pre-planned treatment for a pre-existing condition that is unrelated to the indication under study and has not worsened since signing the information consent
  - Social reasons and respite care in the absence of any deterioration in the patient’s general condition
  - Treatment on an emergency outpatient basis for an event not fulfilling any of the definitions of a SAE given above and not resulting in hospital admission
- Is medically significant, e.g. defined as an event that jeopardizes the patient or may require medical or surgical intervention to prevent one of the outcomes listed above

Medical and scientific judgment should be exercised in deciding whether other situations should be considered serious reactions, such as important medical events that might not be immediately life threatening or result in death or hospitalization but might jeopardize the patient or might require intervention to prevent one of the other outcomes listed above. Such events should be considered as “medically significant”. Examples of such events are intensive treatment in an emergency room or at home for allergic bronchospasm, blood dyscrasias or convulsions that do not result in hospitalization or development of dependency or abuse (please refer to the [ICH-E2D Guidelines 2004](#_bookmark33)).

All malignant neoplasms will be assessed as serious under “medically significant” if other seriousness criteria are not met and the malignant neoplasm is not a disease progression of the study indication.

Any suspected transmission via a medicinal product of an infectious agent is also considered a serious adverse reaction.

All reports of intentional misuse and abuse of the product are also considered serious adverse event irrespective if a clinical event has occurred.

### SAE reporting

To ensure patient safety, every SAE, regardless of causality, occurring after the patient has provided informed consent and until 30 days following the last administration of study treatment if there are post-treatment follow-up visits with no required procedures must be reported to Novartis safety within 24 hours of learning of its occurrence. Detailed instructions regarding the submission process and requirements are provided to each site.

**Screen failures (e.g. a patient who is screened but is not treated)**

SAEs occurring after the patient has provided informed consent until the time the patient is deemed a Screen Failure must be reported to Novartis.

**Treated Patients**

SAEs collected between time patient signs ICF until 30 days after the patient has discontinued or stopped study treatment must be reported to Novartis.

All follow-up information for the SAE including information on complications, progression of the initial SAE and recurrent episodes must be reported as follow-up to the original episode within 24 hours of the investigator receiving the follow-up information. An SAE occurring at a different time interval or otherwise considered completely unrelated to a previously reported one must be reported separately as a new event.

If the SAE is not previously documented in the Investigator’s Brochure (new occurrence) and is thought to be related to the study treatment, a CMO & PS Department associate may urgently require further information from the investigator for health authority reporting. Novartis may need to issue an Investigator Notification (IN) to inform all investigators involved in any study with the same study treatment that this SAE has been reported.

Suspected Unexpected Serious Adverse Reactions (SUSARs) will be collected and reported to the competent authorities and relevant ethics committees in accordance with EU Guidance 2011/C 172/01 or as per national regulatory requirements in participating countries.

Any SAEs experienced after the 30 day period following the last administration of study treatment should only be reported to Novartis Safety if the investigator suspects a causal relationship to study treatment.

### Pregnancy reporting

To ensure patient safety, each pregnancy occurring after signing the informed consent form must be reported to Novartis within 24 hours of learning of its occurrence. The pregnancy should be followed up to determine outcome, including spontaneous or voluntary termination, details of the birth, and the presence or absence of any birth defects, congenital abnormalities, or maternal and/or newborn complications.

Pregnancy should be recorded and reported by the investigator to the Novartis Chief Medical Office and Patient Safety (CMO&PS). Pregnancy follow-up should be recorded on the same form and should include an assessment of the possible relationship to the investigational treatment of any pregnancy outcome. Any SAE experienced during pregnancy must be reported.

### Reporting of study treatment errors including misuse/abuse

Study treatment errors and uses outside of what is foreseen in the protocol will be recorded on the appropriate CRF irrespective of whether or not it is associated with an AE/SAE and reported to Safety (only if associated with an SAE).

Misuse or abuse will be collected and reported in the safety database irrespective of it being associated with an AE/SAE within 24 hours of Investigator’s awareness.

**Table 10-1 Guidance for capturing the study treatment errors including misuse/abuse**

**Treatment error type Document in Dosing**

**CRF (Yes/No)**

**Document in AE eCRF**

**Complete SAE form**

Unintentional study treatment error

Yes Only if associated

with an AE

Only if associated with an SAE

Misuse/Abuse Yes Yes Yes, even if not associated with a SAE

For more information on AE and SAE definition and reporting requirements, please see the respective sections.

## Additional Safety Monitoring

Not applicable

# Data collection and database management

## Data collection

Data not requiring a separate written record will be defined in the protocol and the Assessment Schedule ([Table 8-1](#_bookmark16)) and can be recorded directly on the CRFs. All other data captured for this study will have an external originating source (either written or electronic) with the CRF not being considered as source.

All data should be recorded, handled and stored in a way that allows its accurate reporting, interpretation and verification.

Designated investigator staff will enter the data required by the protocol into the electronic Case Report Forms (eCRF). The eCRFs are built using fully validated secure web-enabled software that conforms to 21 CFR Part 11 requirements. Automatic validation programs check for data discrepancies in the eCRFs, allow modification and/or verification of the entered data by the investigator staff. Investigator site staff will not be given access to the EDC system until they have been trained.

The investigator/designee is responsible for assuring that the data recorded on CRFs (entered into eCRF) is complete, accurate, and that entry and updates are performed in a timely manner. The Investigator must certify that the data entered is complete and accurate.

After the final database lock, the investigator will receive copies of the patient data for archiving at the investigational site.

All data should be recorded, handled and stored in a way that allows accurate reporting, interpretation, and verification.

## Database management and quality control

Novartis personnel (or designated CRO) will review the data entered by investigational staff for completeness and accuracy. Electronic data queries stating the nature of the problem and requesting clarification will be created for discrepancies and missing values. Queries are sent to the investigational site via the EDC system. Designated investigator site staff are required to respond promptly to queries and to make any necessary changes to the data.

Concomitant treatments and prior medications entered into the database will be coded using the WHO Drug Reference List, which employs the Anatomical Therapeutic Chemical classification system. Medical history/current medical conditions and adverse events will be coded using the Medical Dictionary for Regulatory Activities (MedDRA) terminology.

Once all of the necessary actions have been completed and the database has been declared to be complete and accurate, it will be locked and made available for data analysis/moved to restricted area to be accessed by the independent programmer and statistician. Any changes to the database after that time can only be made after written agreement by Novartis development management.

## Site monitoring

Before study initiation, at a site initiation visit or at an investigator’s meeting, a Novartis or delegated CRO representative will review the protocol and data capture requirements (i.e. eCRFs) with the investigators and their staff. During the study, Novartis employs several methods of ensuring protocol and GCP compliance and the quality/integrity of the sites’ data. The field monitor will visit the site to check the completeness of patient records, the accuracy of data capture / data entry, the adherence to the protocol and to Good Clinical Practice, the progress of enrollment, and to ensure that study treatment is being stored, dispensed, and accounted for according to specifications. Key study personnel must be available to assist the field monitor during these visits. Continuous remote monitoring of each site’s data may be performed by a centralized Novartis or delegated CRO/CRA organization. Additionally, a central analytics organization may analyze data & identify risks & trends for site operational parameters, and provide reports to Novartis clinical teams to assist with trial oversight.

The investigator must maintain source documents for each patient in the study, consisting of case and visit notes (hospital or clinic medical records) containing demographic and medical information, laboratory data, electrocardiograms, and the results of any other tests or assessments. All information on CRFs must be traceable to these source documents in the patient’s file. The investigator must also keep the original informed consent form signed by the patient (a signed copy is given to the patient).

The investigator must give the monitor access to all relevant source documents to confirm their consistency with the data capture and/or data entry. Novartis monitoring standards require full verification for the presence of informed consent, adherence to the inclusion/exclusion criteria, documentation of SAEs and of data that will be used for all primary variables. Additional checks of the consistency of the source data with the CRFs are performed according to the study- specific monitoring plan. No information in source documents about the identity of the patients will be disclosed.

# Data analysis and statistical methods

Any data analysis carried out independently by the investigator should be submitted to Novartis before publication or presentation.

## Analysis sets

The safety analysis set will include all patients that received one (1) IVT injection of brolucizumab 6 mg. All analyses will be performed on the Safety Set unless otherwise specified.

## Subject demographics and other baseline characteristics

Demographic and Baseline characteristics will be summarized descriptively. Categorical data will be presented as frequencies and percentages. For continuous data, mean, standard deviation, median, minimum and maximum will be presented*.*

Relevant medical histories and current medical conditions at Baseline will be summarized by system organ class and preferred term. Separate tables will be presented for ocular and non-ocular histories and conditions.

For selected characteristics, listings will be presented by patient.

## Treatments

Concomitant medications and significant non-drug therapies prior to and after the start of the study treatment will be listed and summarized according to the Anatomical Therapeutic Chemical (ATC) classification system.

## Analysis of the primary endpoint(s)

The primary objective of this study is to collect information on ECG after intravitreal injection of brolucizumab 6 mg in patients with nAMD.

### Definition of primary endpoint(s)

The primary endpoints include the following:

- Incidence between 20 and 24 h post-injection of clinically relevant treatment emergent changes in HR, PR, QRS, and QTc (heart rate corrected QT using Fridericia’s formula, QTcF) interval (ms)

### Statistical model, hypothesis, and method of analysis

There are no formal hypotheses in this study.

Incidences between 20 and 24 h post-injection of clinically relevant treatment emergent changes in HR, PR, QRS, and QTc interval will be listed by patient.

The frequency and proportion of patients with change from baseline in QTcF ≥ 30 ms and

≥ 60 ms between 20 and 24 h post-injection will be summarized. Listings of patients with change from baseline in QTcF ≥ 30 ms and ≥ 60 ms between 20 and 24 h post-injection will be provided.

The frequency and proportion of patients with QTcF > 450, 480, and 500 ms between 20 and 24 h post-injection will be summarized. Listings of patients with QTcF > 450, 480, and 500 ms between 20 and 24 h post-injection will be provided separately.

Holter ECG data between 20 and 24 h post-injection will be plotted by patient, and abnormalities will be flagged.

Further details on analyses of primary endpoints will be provided in the SAP.

### Handling of missing values/censoring/discontinuations

There will be no imputation of missing data. Only observed data will be used for the analyses.

### Sensitivity and Supportive analyses

There will be no sensitivity analyses.

## Analysis of secondary endpoints

The secondary endpoints are ocular and non-ocular AEs (including clinically relevant ECG abnormalities) until the end of the study.

### Safety endpoints

A treatment-emergent adverse event is defined as any adverse event that develops after initiation of the study treatment or any event already present that worsens following exposure to the study treatment, until the end of study. Only treatment-emergent adverse events will be presented in the summary tables.

Adverse events will be coded using the MedDRA dictionary and presented by system organ class (SOC) and preferred term (PT). AEs will be presented based on the number and percentage of patients with at least one AE in the category of interest. Separate tables will be provided for ocular events in the study eye and fellow eye and non-ocular events (including clinically relevant ECG abnormalities).

Patient listings of all adverse events will be provided. Deaths and other serious or clinically significant non-fatal adverse events will be listed separately.

## Interim analyses

Not applicable.

## Sample size calculation

### Primary endpoint(s)

The PMDA of Japan requested a sample size of approximately 10 to 15 patients for this study. No sample size calculations were performed.

# Ethical considerations and administrative procedures

## Regulatory and ethical compliance

This clinical study was designed and shall be implemented, executed and reported in accordance with the ICH Harmonized Tripartite Guidelines for Good Clinical Practice, with applicable local regulations (including European Directive 2001/20/EC, US CFR 21), and with the ethical principles laid down in the Declaration of Helsinki.

## Responsibilities of the investigator and IRB/IEC

Before initiating a trial, the investigator/institution must obtain approval/favorable opinion from the Institutional Review Board/Independent Ethics Committee (IRB/IEC) for the trial protocol, written informed consent form, consent form updates, patient recruitment procedures (e.g., advertisements) and any other written information to be provided to patients. Prior to study start, the investigator is required to sign a protocol signature page confirming his/her agreement to conduct the study in accordance with these documents, all of the instructions and procedures found in this protocol, and to give access to all relevant data and records to Novartis monitors, auditors, Novartis Quality Assurance representatives, designated agents of Novartis, IRBs/IECs, and regulatory authorities as required. If an inspection of the clinical site is requested by a regulatory authority, the investigator must inform Novartis immediately that this request has been made.

## Publication of study protocol and results

The protocol will be registered in a publicly accessible database such as clinicaltrials.gov and as required in EudraCT. In addition, after study completion (defined as last patient last visit) and finalization of the study report, the results of this trial will be and posted in a publicly accessible database of clinical trial results, such as the Novartis clinical trial results website and all required Health Authority websites (e.g. Clinicaltrials.gov, EudraCT, etc.).

For details on the Novartis publication policy including authorship criteria, please refer to the Novartis publication policy training materials that were provided at the trial investigator training.

## Quality Control and Quality Assurance

Novartis maintains a robust Quality Management System (QMS) that includes all activities involved in quality assurance and quality control, to ensure compliance with written Standard Operating Procedures as well as applicable global/local GCP regulations and ICH Guidelines.

Audits of investigator sites, vendors, and Novartis systems are performed by auditors, independent from those involved in conducting, monitoring or performing quality control of the clinical trial. The clinical audit process uses a knowledge/risk based approach.

Audits are conducted to assess GCP compliance with global and local regulatory requirements, protocols and internal SOPs, and are performed according to written Novartis processes

# Protocol adherence

This protocol defines the study objectives, the study procedures, and the data to be collected on study participants. Additional assessments required to ensure safety of patients should be administered as deemed necessary on a case by case basis. Under no circumstances including incidental collection is an investigator allowed to collect additional data or conduct any additional procedures for any purpose involving any investigational drugs under the protocol, other than the purpose of the study. If despite this interdiction prohibition, data, information, observation would be incidentally collected, the investigator shall immediately disclose it to Novartis and not use it for any purpose other than the study, except for the appropriate monitoring on study participants.

Investigators ascertain they will apply due diligence to avoid protocol deviations. If an investigator feels a protocol deviation would improve the conduct of the study this must be considered a protocol amendment, and unless such an amendment is agreed upon by Novartis and approved by the IRB/IEC and health authorities, where required, it cannot be implemented.

## Protocol amendments

Any change or addition to the protocol can only be made in a written protocol amendment that must be approved by Novartis, health authorities where required, and the IRB/IEC prior to implementation.

Only amendments that are required for patient safety may be implemented immediately provided the health authorities are subsequently notified by protocol amendment and the reviewing IRB/IEC is notified.

Notwithstanding the need for approval of formal protocol amendments, the investigator is expected to take any immediate action required for the safety of any patient included in this study, even if this action represents a deviation from the protocol. In such cases, Novartis should be notified of this action and the IRB/IEC at the study site should be informed according to local regulations.

# References

References are available upon request

Blinder KJ, Bradley S, Bressler NM, et al (2003) Effect of lesion size, visual acuity, and lesion composition on visual acuity change with and without verteporfin therapy for choroidal neovascularization secondary to age-related macular degeneration: TAP and VIP report no. 1. Am J Ophthalmol. 136:407–18.

Bloch SB, Larsen M, Munch IC (2012) Incidence of legal blindness from age-related macular degeneration in Denmark: Year 2000 to 2010. Am J Ophthalmol. 153:209-13.

Campbell JP, Bressler SB, Bressler NM (2012) Impact of availability of anti-vascular endothelial growth factor therapy on visual impairment and blindness due to neovascular age- related macular degeneration. Arch Ophthalmol. 130:794-5.

Ferris FL, Fine SL, Hyman L (1984) Age-related macular degeneration and blindness due to neovascular maculopathy. Arch Ophthalmol. 102:1640-42.

ICH E2D (2004) Clinical Safety Data Management: Definitions and Standards for expedited reporting. Accessed from: <http://www.ema.europa.eu/docs/en_GB/document_library/Scientific_guideline/2009/09/WC5> 00002807.pdf Accessed on: 02Dec2016.

Kawasaki R, Yasuda M, Song S, et al (2010) The prevalence of age-related macular degeneration in Asians: A systematic review and meta-analysis. Ophthalmology. 117:921-27.

Malik M, Garnett CE, Zhang J (2010) Thorough QT Studies: Questions and Quandaries. Drug Saf. 33(1):1-14.

Lim LS, Mitchell P, Seddon JM, et al (2012) Age-related macular degeneration. Lancet. 379:1728-38.

Miller JW (2013) Age-Related Macular Degeneration Revisited – Piecing the Puzzle: The LXIX Edward Jackson Memorial Lecture. Am J Ophthalmol. 155:1-35.

Rein DB, Wittenborn BS, Zhang X, et al (2009) Vision Health Cost-Effectiveness Study Group. Forecasting age-related macular degeneration through the year 2050. Arch Ophthalmol. 127:533-40.

Shah AR, Del Priore LV (2007) Progressive visual loss in subfoveal exudation in age-related macular degeneration: a meta-analysis using Lineweaver-Burke plots. Am J Ophthalmol.

143:83-89.

Shah AR, Del Priore LV (2009) Natural history of predominantly classic, minimally classic and occult subgroups in age-related macular degeneration. Am J Ophthalmol. 116:1901-07.

Smith W, Assink J, Klein R, et al (2001) Risk factors for age-related macular degeneration: Pooled findings from three continents. Ophthalmology. 108:697-704.

Spilsbury K, Garrett KL, Shen WY, et al (2000) Over expression of vascular endothelial growth factor (VEGF) in the retinal pigment epithelial leads to the development of choroidal neovascularization. Am J Pathol. 157:135-44.

Wong WL, Su X, Li X, et al (2014) Global prevalence of age-related macular degeneration and disease burden projection for 2020 and 2040: a systematic review and meta-analysis. Lancet Glob Health;2:e106-16.
